# Norovirus: Facts and Reflections from Past, Present, and Future

**DOI:** 10.3390/v13122399

**Published:** 2021-11-30

**Authors:** Yalda Lucero, David O. Matson, Shai Ashkenazi, Sergio George, Miguel O’Ryan

**Affiliations:** 1 Microbiology and Mycology Program, Institute of Biomedical Sciences, Faculty of Medicine, Universidad de Chile, Santiago 8380453, Chile; ylucero@gmail.com (Y.L.); sergio.george@gmail.com (S.G.); 2 Hospital Dr. Roberto del Río Hospital, Department of Pediatrics and Pediatric Surgery (Northern Campus), Faculty of Medicine, Universidad de Chile, Santiago 8380418, Chile; 3 Clínica Alemana de Santiago, Faculty of Medicine, Universidad del Desarrollo-Clínica Alemana, Santiago 7650568, Chile; 4 Eastern Shore Health Department, Virginia Department of Public Health, Accomack County, VA 23301, USA; wrenpt@gmail.com; 5 Adelson School of Medicine, Ariel University, Ariel 40700, Israel; shai.ashkenazi7@gmail.com; 6 Department of Pediatrics A, Schneider Children’s Medical Center, Petach Tikva 49202, Israel

**Keywords:** Norovirus, epidemiology, prevention, vaccine

## Abstract

Human Norovirus is currently the main viral cause of acute gastroenteritis (AGEs) in most countries worldwide. Nearly 50 years after the discovery of the “Norwalk virus” by Kapikian and colleagues, the scientific and medical community continue to generate new knowledge on the full biological and disease spectrum of Norovirus infection. Nevertheless, several areas remain incompletely understood due to the serious constraints to effectively replicate and propagate the virus. Here, we present a narrated historic perspective and summarize our current knowledge, including insights and reflections on current points of interest for a broad medical community, including clinical and molecular epidemiology, viral–host–microbiota interactions, antivirals, and vaccine prototypes. We also include a reflection on the present and future impacts of the COVID-19 pandemic on Norovirus infection and disease.

## 1. Introduction

For over 50 years now, the scientific and medical community have been gathering knowledge on the full biological and disease spectrum associated with human caliciviruses, which are currently grouped into two genera: the Norovirus and Sapovirus. This review will focus upon Norovirus (NoV), which is currently the most commonly detected human pathogen of the family. We will focus upon key historical aspects and state-of-the-art knowledge that may be of interest to a broad medical readership and we will propose what we believe are the main challenges for better control of NoV-associated disease.

## 2. The Past

### 2.1. Virus Discovery

**Figure 1:**
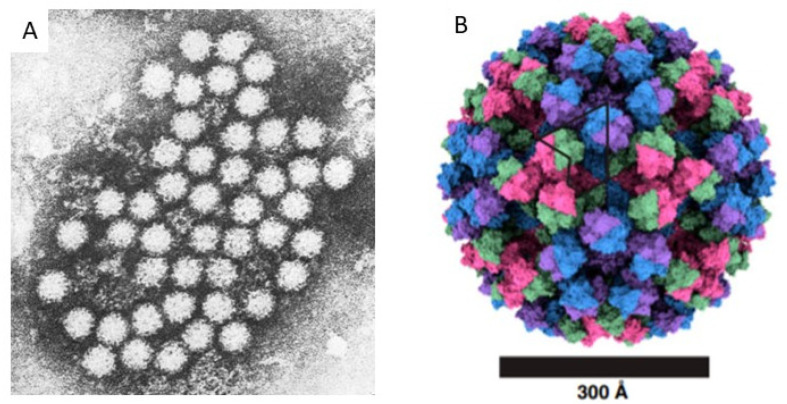
Electron microscopy images of Noroviruses. (**A**). Original image of small round structured viruses, visualized by Kapikian in stool samples from acute gastroenteritis cases, by immune electron microcopy [7]. (**B**). High resolution cryo-electron microscopy of a GII.4 Norovirus particle [9].

In 1970, Yow and Melnick, with others [1], reported that no viruses could be imputed to be frequent causes of acute gastroenteritis (AGE) in children. In a several-year study, they, like others, found the bacteria enteropathogenic *E. coli*, *Salmonella* spp., and *Shigella* spp. more often in hospitalized children with AGE than among control children. However, two breakthrough technologies led to the discovery of many novel causes of AGE, including viruses. One such technology was that of the Nobel Prize-winning competition radioimmunoassay of Yalow and Berson [2,3], in which antibody molecules reacted with insulin to form antibody–target complexes that could be quantified. The other, was transmission electron microscopy (TEM) [4], which allowed samples to be magnified up to 70,000 times. On the other hand, at this stage, no animal or cell line model was available able to replicate infection. Thus, research in human volunteers was the only model to advance in characterizing and demonstrating the pathogenic role of this agent in AGE [5,6]. Kapikian was able to visualize NoV particles combining the application of these two technologies, after creating antibody–target complexes (Figure 1) [7]. His original specimens came from an AGE outbreak at an elementary school in Norwalk, Ohio. He studied specimens from people affected in the outbreak and from human volunteers who consented to swallow inoculates from that outbreak. The following is how Kapikian described his original discovery:

“In 1972, a 27 nm virus-like particle was discovered by use of immune electron microscopy (IEM) in an infectious stool filtrate derived from an outbreak of gastroenteritis in an elementary school in Norwalk, Ohio. IEM enabled the direct visualization of antigen-antibody interaction, as the particles were aggregated and coated by specific antibodies. This allowed the recognition and identification of a 27 nm virus-like particle that did not have a distinctive morphology, was low-titered, and was among the smallest viruses known. Serum antibody responses to the 27 nm particle were demonstrated in key individuals infected under natural or experimental conditions; this and other evidence suggested that this virus-like particle was the etiologic agent of the Norwalk gastroenteritis outbreak. The fastidious 27 nm Norwalk virus is now considered to be the prototype strain of a group of non-cultivatable viruses that are important etiologic agents of epidemic gastroenteritis in adults and older children”.

[8]

Filtered and treated stools administered to human volunteers confirmed that the causative agent was small (<36 nm), nonenveloped, and relatively heat stable [10]. Attempts to in vitro or in vivo passage of the virus, except in human volunteers, were unsuccessful for a long time [11].

### 2.2. The Evolution from Antigenic to Molecular Studies

After Kapikian’s success at virus visualization in 1972, the next major achievement occurred in 1979, when Greenberg and colleagues [12], working with collaborators at the Centers for Disease Control, reported that approximately one-third of outbreaks of AGEs in adults, with no known bacterial or parasitic cause were likely caused by Norwalk-like viruses. This observation was confirmed by many reports in other latitudes.

The first images of NoV provided by Kapikian [7] showed a uniformly round, rough-surfaced particle. Greenberg and colleagues [13] directly assessed proteins from stool specimens containing Norwalk virus and found an approximately 59,000 mW protein, sometimes accompanied by an approximately 30,000 mW protein. These findings were clarified when the genome was sequenced in 1989 by Jiang et al. [14]; one of the open reading frames (ORF) predicted from the sequence encoded a protein with a molecular weight of ~58,000 mW and virus-like particles (VLPs) were created in a baculovirus expression system [15]. The 59,000 mW protein in stool specimens was determined to be the same as the 58,000 protein in the VLPs from the expressed ORF [16]. The virion consists of 180 of these molecules formed in an icosahedron, appearing to be a round structure at resolutions lower than cryo-electron microscopy (Figure 1) [7,9,17]. Further details of the structure are beyond the scope of this review.

As more and more small viruses were characterized, many were recognized to be nearly round, with a single-stranded RNA genome, to have a unique organization of protein-encoding elements, to have a variable role in human and animal disease, and many were found to be causative agents of AGE [18,19,20].

In the 1990’s with the development of enzyme-linked immunosorbent assay (ELISA), further knowledge as to the epidemiologic role of Norwalk-like viruses epidemiology was possible. Paradoxically, these agents were then identified as a major cause of food-borne diarrhea in adults but were infrequently found to be a cause of AGE in children. The largest systematic assessment of hospitalized children found NoV in 7.1% of AGE cases [21]. At the time, it appeared that there may be a difference in pathogenesis between children and adults infected with the same strains, which could explain the differing conclusions. Later, antigenic diversity and the low sensitivity of ELISA tests were determined to be underlying this apparent discrepancy.

Since the 2000s, as genome characterization of many more NoV strains increased, and more investigators shared their information of NoV sequences from different genomic regions, a wide variability became evident and recombination among strains was recognized [22,23], as will be discussed further below.

## 3. The Present

### 3.1. Clinical Epidemiology

Given the progress made in its detection by sensitive molecular diagnostic techniques and the introduction of the rotavirus vaccine, NoV has emerged in recent years as a leading causative agent of AGE in most locations and age groups [24,25,26,27]. It is associated with nearly 20% of all acute diarrheal cases globally, and with an estimated 685 million episodes and 212,000 deaths annually [24,28,29]. Each year in the United States, NoV causes 19 to 21 million illnesses, including 900 deaths, 103,000 hospitalizations, 460,000 emergency department visits, and 2.6 million outpatient visits [30]. It has been identified as a relevant cause of acute endemic diarrhea in children under 5 years of age as well as the primary agent of AGE outbreaks affecting individuals of all ages.

Among 148,867 patients with AGE reported in 178 publications, the overall detection rate of NoV was 17% (95% CI: 15–18%) [31]. In a study comparing 12,531 children with AGE and 11,954 control subjects from 45 countries across the world, the pooled detection rate of NoV was 17.7% (95% CI: 16.3–19.2%) and 6.7% (95% CI: 5.1–8.8%), respectively, giving a pooled OR of 2.7 for the association of NoV infection and AGE (95% CI: 2.2–3.4) [24].

NoV infections are common both in developed and developing countries, affecting all socioeconomic classes; nevertheless, socioeconomic status is related to the risk of acquisition and severity of NoV infections. A systematic review has demonstrated that the detection rate of NoV in AGE episodes decreased from 18% (95% CI: 16–20%) for upper middle-income countries to 15% (13–18%) and 6% (3–10%) for lower middle- and low-income countries, respectively [31]. In another review, NoV was detected in 15% (95% CI 15–16%) of children with AGE in low–middle-income countries (vs. 8% of healthy controls) and 11% (95% CI 10–12%) of those in low-income countries (vs. 9% of asymptomatic controls) [32]. Yet, birth cohort studies conducted in low- and middle-income countries have demonstrated that 66–90% of the children experienced at least one NoV infection and 30–70% experienced NoV-associated AGE in early childhood, with an estimated incidence for NoV-associated AGE of 14 to 66 per 100 child years [25]. Male predominance has been often reported [24,33], with the highest rates of NoV AGE during winter or rainy seasons [34,35,36].

Endemic NoV gastroenteritis typically affects young children under 5 years of age [24,25,37]. A recent meta-analysis found that the highest frequency of NoV infection occurred in infants less than 12 months of age [24]. A second study of hospitalized patients in Indonesia found the highest frequency of NoV infection in those under 24 months of age [34]. In a summary of 10 community-based birth cohort studies, NoV-associated AGE was relatively uncommon in infants under 6 months of age [25].

Foodborne outbreaks are a significant problem for public health, causing outbreaks in otherwise healthy individuals that last 1 to 4 days [26,38]. NoV is the most common cause of such outbreaks, accounting for about 50% of all-cause events worldwide and >90% of viral AGE outbreaks. According to a 2015 World Health Organization report, NoV was the primary cause of foodborne illnesses globally, accounting for 124,803,946 cases and 34,929 deaths, indicating a fatality rate of 0.028 [39]. Similarly, in the United States, NoV caused the highest number of cases of foodborne outbreaks, with an increase from 2563 cases in 1998 to 5135 in 2017, including 4 deaths [40].

Foodborne outbreaks can be caused either by contamination at the source or during food handling, processing, or serving. A systematic review of NoV-attributed outbreaks between January 2003 and July 2017 identified 27 publications that met the definition of confirmed outbreaks caused by food contaminated at source and 47 that met the criteria for outbreaks caused by food handlers [38]. Of all studies, the food type most commonly implicated in outbreaks was seafood (61%); within this group, 89% of outbreaks were associated with oysters and 10% with shellfish. Other commonly infected foods included berries, other fruits or vegetables, salads, and occasionally processed meats [38,40,41]. Outbreaks attributed to food handling involved a diverse range of foodstuffs and occurred in multiple settings, including restaurants (most common), events with catering services such as weddings, and military units [38].

### 3.2. Molecular Epidemiology

The NoV genome comprises a positive-sense, single-stranded, polyadenylated RNA of approximately 7700 nucleotides with 3 open reading frames (ORFs 1–3) [17,24]. Based upon phylogenetic analysis, human NoVs are classified into ten genogroups designated GI to GX, and two tentative genogroups—GNA1 and GNA2—have also been described. Most strains implicated in human disease belong to three genogroups: GI, GII, and GIV [42]. The genogroups are further classified into genotypes based on the capsid protein 1 (VP1) sequence, encoded by ORF2 with more than 45 variants described. Although a consensus genotyping based on VP1 (G-type) is the most widely used, a binomial nomenclature system considering also the RNA polymerase (RdRp) sequence, encoded by ORF1 (P-type), has been proposed, as recombination occasionally occurs at the ORF1/ORF2 junction of the viral genome [42]. In addition, variants (sub-genotypes) are determined by sequence analysis of highly variable regions of the ORF2 and named according to the location and year where they were first described.

Molecular studies have confirmed the complex genomic diversity of circulating NoVs, which vary by location, age, and sometimes the clinical presentation and other variables, with the emergence of new genotypes or variants every few years, that replace previously predominant strains [24,25,31,43,44]. A recent meta-analysis of articles published from January 2015 to May 2020 explored the global distribution of genotypes of NoV infections among children with AGE [24]. Of the 123 studies included in the analysis, which included 120,531 individuals with AGE, most of the NoVs belonged to the GII genogroup (92.9%; 95% CI: 90.6–94.6%), followed by GI, with a significantly lower prevalence (6.7%; 95% CI: 5.2–8.5%) (*p* < 0.0001). A total of 31 genotypes were detected: 12 from the GI genogroup and 19 from the GII genogroup. The most prevalent genotypes were GII.4 (59.3%, 95% CI: 53.4–64.9%), GII.3 (14.9%, 95% CI: 10.6–20.5%), and GII.12 (5.1%, 95% CI: 2.9–8.7%) [24]. The GII.4 strain has consistently been identified as the predominant genotype, with new GII.4 strains emerging every 2–5 years, often replacing previously predominant variants. Between 2000 and 2012, a systematic review of 51 publications identified the following variants as dominant: GII.4/2002, GII.4/2004, GII.4/2006b, and GII.4/2008 [45]. The pandemic GII.4 variant, Sydney 2012, was first reported in early 2012 and soon became the predominant circulating strain globally, replacing those previously described [24,46,47,48].

Selected recent publications on the genotype distribution of human NoVs in various locations and the main variants identified, focused on both sporadic episodes and outbreaks, are detailed in Table 1. These studies differed in various aspects, such as their design (prospective, sometimes population-based, vs. retrospective analysis), age of participants, sporadic cases vs. outbreak-based studies, and/or hospitalized vs. community cases [33,34,43,48,49,50,51,52,53,54,55,56]. Although the relative prevalence of NoV genotypes varied among studies and locations, genotype GII.4 was the most common among all studies. Recombinant strains and GII.4 variants were reported frequently [24,32,33,43,45,46,47,49,54,56] and most of the studies documented changes in the dominant strains during the study period [33,34,35,45,53], with the emerging strain occasionally causing more severe disease [33].

Some differences in the epidemiologic features of the genotypes were observed. For example, in a relatively small study in Qatar, while NoV GII.4 was reported in all age groups, genotype GII.3 infections were more common in children <1 year of age [33]. In Japan, patients infected with genotypes GII.2 and GII.6 were younger than those infected with GII.4 [53]. Geographic differences among circulating genotypes, as well as the viral load associated with disease, were identified. Studies of NoV outbreaks in China showed that GII.2 outbreaks mainly occurred in day care centers, elementary schools, and high schools and were primarily transmitted mainly through person-to-person contact, while GII.4/2012 Sydney outbreaks frequently occurred in colleges and were primarily associated with foodborne transmission [46]. In Japan, outbreaks of the genotype GII.17 occurred frequently as foodborne [53]. Vomiting was more commonly reported by patients infected by GII.2 and GII.17 outbreaks compared with outbreaks associated with the GII.4 genotype [45]. Despite the above, well defined epidemiological or clinical patterns for specific genotypes have not been identified.

The considerable genomic diversity of human NoVs does not necessarily imply antigenic diversity; indeed, antigenic commonality might be of greater benefit if common antigens are neutralizing, as it was demonstrated for rotaviruses (RoVs), for which monoclonal antibodies were created that neutralized several serotypes. As new genetic diversity is described for NoVs, this experience with RoVs ought to be remembered. However, the pathway for NoV antigenic characterization has been much slower and remains a difficult area of study. The major impediment has been inability to readily replicate the virus in the laboratory. Lacking the ability to study specific- and cross-neutralization in cell culture or the human host has been a fundamental impediment to vaccine development. A recent, major advance in NoV studies has been the successful replication of human NoVs in cell cultures [57,58]; yet, to date, the techniques are cumbersome and specific, indicating that widespread application will not likely occur in the near future.

### 3.3. Transmission and Shedding

Human-to-human transmission of NoV is common, mainly by the fecal–oral route (particularly for the epidemic strain GII.4); although, spread may be enhanced by episodes of vomiting [59]. NoV can efficiently survive in the environment and resists freezing temperatures, heating to 60 °C, and disinfection with chlorine or alcohol—facilitating contamination of food, water, and inanimate surfaces [60]—eventually leading to outbreaks, especially in close settings as daycare centers, schools [46,61], hospitals, military camps, and cruise ships [41,62], among others. NoV is highly contagious due to its very low infectious dose; a single particle has an infection probability of 50%, although a dose–response relationship has been noted, in which individuals exposed to higher numbers of viruses experience a higher infectious rate [34,47,61,63].

NoV shedding in stools is maximum during the first 24 to 48 h after symptoms onset, with a mean duration of four weeks [64,65,66]. In immunocompromised hosts, viral shedding in stools can persist for months following infection [65,67].

### 3.4. Clinical Presentation

Outbreaks and volunteer studies have reported about 30% of asymptomatic infections [68]. In symptomatic patients, after a 24–48 h incubation period, a variable combination of acute watery diarrhea, nausea, vomiting, malaise, abdominal pain, and/or headache, that last 1–4 days, have been reported. Fever is less frequent than in RoV infection.

Among children, gastroenteritis due to NoV had slightly lower severity as compared with RoV-caused gastroenteritis, with a 20% decrease in dehydration [69], although a study in Bangladesh found higher rates of moderate–severe dehydration in NoV gastroenteritis [70]. It should be emphasized that older adults (>60 years) are at increased risk of severe and complicated course of NoV AGE. For example, a decade-long analysis of NoV disease risk has shown that older adults living in long-term care facilities in middle–high- and high-income countries experienced frequent NoV outbreaks, leading to high hospitalization rates of 0.5–6% and case fatality rates of 0.3–1.6% [71]. Likewise, of the elderly individuals hospitalized for NoV infection in Atlanta, Georgia (United States), 36% were admitted to intensive care units [44]. Similarly, healthcare-associated infections, mainly in hospital wards, can occur.

Although NoV infections in AGE are mostly short and self-limited, chronic diarrhea in immunocompromised, malnourished, and elderly patients has been described [72,73,74]. On the other hand, immune-mediated disease may be triggered such as necrotizing enterocolitis in newborns [75,76] and inflammatory bowel disease exacerbations [77,78]. Dysregulation of enteric nervous and immune system may also be associated with the development of post-infectious irritable bowel syndrome [79,80].

NoV has also been associated with compromised extraintestinal function and has been detected in serum and cerebrospinal fluid samples [81,82]. Encephalopathy, seizures, and liver disfunction have been described in this context; however, a causal role is still controversial [83,84,85,86].

### 3.5. NoV and Host Interactions

Until recently, when a human NoV (HNoV) replication model was developed in Zebrafish [87], other animal models were not available, and most of the needed evidence about virus–host relationship came from studies of Murine NoV (MNoV) [88]. MNoV can infect and replicate in several immune cells (including T cells, B cells, dendritic cells, macrophages). A specific tropism for Tuft cells, a chemosensory rare type of intestinal epithelial cell (IEC) of CR6 MNoV, which causes persistent infection, has been described [88,89]. MNoV uses a species-specific proteinaceous receptor (CD300LF) to attach cells [90] through binding sites in protruding (P) domains of the major capsid protein (VP1). This relationship is dynamic and dependent on P-domain conformational changes [91,92], which can be induced by cofactors such as bile acids, which enhance cell-attachment [93,94]. Though initial data suggested the binding of MNoV to host carbohydrates [95], recent studies based on nuclear magnetic resonance spectroscopy show that MNoV P-domains do not bind to surface sialoglycans [96].

The detection of viral non-structural proteins in enterocytes of human adults’ biopsies [97], and the presence of viral non-sense RNA in intestinal enteroendocrine cells from pediatric intestinal biopsies [98], suggest that HuNoV replicates in intestinal cells during infection, which is supported by its ability to replicate in human intestinal enteroids (HIE) [57,99]. While replication of HNoV in B cell lines has been shown [100,101], there is lack of evidence of in vivo replication in immune cells [100]. Though a specific protein receptor for HNoV has not been identified, it binds to histo-blood group antigens (HBGAs) [57], carbohydrates expressed on the surface of red blood cells and mucosal epithelial cells that can be also secreted into saliva and other fluids accordingly, when a functional Fucosyltransferase-2 (FUT2) enzyme is expressed (secretor status). Those individuals who do not express a functional FUT2 (non-secretors) are resistant to specific HNoV genotypes [102]. Given the importance of HBGA in HNoV pathogenesis, and as glycoslylation patterns in Zebrafish differs from humans [103], this point should be considered for HNoV in vivo studies, in this model. Additionally, in vitro studies have shown that bile acids can bind to HNoV and promote its replication [104]. The apparently crucial role of gut microbiota in the initial phase of infection is discussed further below.

Once MNoV replicates within host cells, viral molecules are sensed by several innate-immune receptors (e.g., TLR, RIG-1 and MDA5) to promote a type I Interferon (IFN-I) response that is needed to eradicate infection [105]. In humans, both a proinflammatory and a regulatory cytokine response is seen during the acute phase [106], which may be related to symptomatology [107]. HNoV replication in HIEs systems have provided evidence of the importance of innate immunity in its pathogenesis; while HNoV triggers a chemokine (CXCL10) [99] and IFN-stimulated genes with a predominantly type III IFN response; however, there is a strain-dependent sensibility to endogenous IFN which can explain the presence of globally expanded strains more resistant to innate-immunity signaling (such as GII.4) [108]. Adaptive immune response to MNoV requires both humoral and cellular components to be effective [109]. Cellular response is dependent on CD4 and CD8 T cells [110] and requires an effective acute IFN response to be generated, while an ineffective innate and then cellular response has been described in strain CR6, which generates a persistent infection in mice [111]. In the case of HNoV, while the role of antibodies limiting infection has been described [112], data on the cellular response is still limited [113]. However, the ability to cause chronic diarrhea in humans lacking cellular immunity [114] suggests that this component is also necessary to limit the infection.

The association between HNoV infection and later onset of irritable bowel syndrome [80] and inflammatory bowel disease (IBD) [115] has been described. Although evidence from MNoV suggests that infections can trigger IBD in genetically susceptible mice [116], currently there is a gap in our knowledge as to the relevance of this association in humans and the immunological mechanisms involved.

### 3.6. NoV-Gut Microbiota Interactions

A complex relationship between NoV infections and the gut microbiota has been described. Antibiotic-treated mice with subsequent microbiota depletion become resistant to infection with strains associated with both acute (MNV-1) [111] and persistent (CR6) [117] MNoV infections, which suggests the crucial role of the microbiota in promoting infection. Secretory IgA is needed for efficient MNoV infection in mice [118], which is promoted by a functional gut microbiota. Furthermore, antibiotic treatment reduces the number of colonic Tuft cells [119]; this is relevant as the microbiota may play a role in promoting tuft cells; however, this association requires further exploration. HNoV can bind to HBGAs-like molecules in gut bacteria, which stimulates viral replication and resistance to heat stress [120]. On the other hand, the gut microbiota can promote an antiviral host response and limit infection. Specific components of the microbiota, e.g., *Lactobacillus* [121] and poly-γ-glutamic acid from *Bacillus* spp. [122] promote an IFN-I response and limit MNoV infections. The abundance of specific microbiota components (*Ruminococcus* and *Faecalibacterium*) in healthy adults is inversely associated with salivary anti-NoV IgA levels [123], suggesting that these specific bacteria are associated with lower susceptibility to NoV infections. A recently published NoV challenge study in adults showed that the pre-infection microbiota of subjects who developed an asymptomatic infection was enriched in *Bacteroidetes* and depleted in *Clostridia*, compared with the symptomatic subjects [124]. Larger prospective cohort studies are needed to elucidate factors involved in the gut microbiota composition-determined predisposition or protection against NoV infections. Finally, the effect of NoV infection on gut microbiota composition has been a matter of active research. MNoV causes a strain-specific effect on gut microbiota composition, as MNV-1 causes significant alterations [125] not found in persistent CR6 infection [126]. In adult humans, a subset of HNoV-infected adults showed a significant change in gut microbiota characterized by an increase in Proteobacteria and a decrease in Bacteroidetes compared with non-infected controls [127]. In children, viral AGE (including HNoV-caused diarrhea) is associated with changes in gut microbiota, including a decrease in alpha-diversity (which tends to be less significant in HNoV compared with RoV-caused diarrhea), and variable changes in specific taxa according to different studies [128,129,130]. In summary, there is a tripartite relationship between NoV, the host, and the host’s gut microbiota, and details about mechanisms involved, and their potential uses in preventive or therapeutical interventions (e.g., microbiota-related response to vaccines or probiotics) are the subjects of ongoing research.

### 3.7. Antivirals: Not There Yet

Although the traditional NoV therapy has been supportive care, mainly hydration, several antivirals have been explored during the past decades. Virus-directed agents that have been proved included receptor blockers (citrate), protease inhibitors, and nucleoside and non-nucleoside RdRp inhibitors. TLR agonists have been explored to stimulate host immune response. Nitazoxanide has also been tested but its mechanism of action is unknown. These compounds are still in pre-clinical stages. For a comprehensive review in this topic, read [131].

The antiviral would have to not only be safe, but also effective in reducing disease and transmission quickly, because of the short clinical course and high transmission rate of this agent. On the other hand, NoV tends to be under-reported and unrecognized because detection is usually not included in routine workout of AGEs and this point should be improved if an etiological treatment is proposed. Closed environments, such as hospitals, nursing homes for elder individuals, and possibly in military scenarios, where NoV outbreaks could pose significant operational interference, seem ideal first testing sites for candidate antiviral agents.

### 3.8. Path to NoV Vaccine Development

Given that NoV is the leading cause of AGEs globally, that it is associated with significant morbidity and mortality, and that there is no specific anti-viral therapy, preventing NoV infections through vaccination is a high priority [25,132].

However, the development of a NoV vaccine has faced significant challenges. These include the genetic diversity of NoVs with the periodic appearance of new mutants and recombinants, limited knowledge on correlates of protection, the absence of an effective cell line to cultivate the virus, and the absence of an easily used and appropriate animal model. Community-based longitudinal birth cohort studies on NoV infection with genotypic characterization, mainly those conducted in Peru, India, and Bangladesh, shed light on a relatively high (86–100%) homotypic protection after natural infection, namely protection from repeated infections by the same NoV genotype [25]. Along the same line, serum specimens collected from children who had been hospitalized because of NoV AGE contained neutralizing IgG antibodies against homologous GII.4 genotypes, with genotype-specific seroconversion [133]. The duration of protection and the level of heterotypic protections have not yet been fully elucidated.

Several avenues for developing a NoV vaccine have been explored during the past years. Table 2 summaries the current status of these candidates. In summary, the vaccines most advanced in clinical trials—which most likely will “see the light” within the next 5 years—are those based on virus-like particles (VLPs). These candidates, two of which have made it to advanced human trials, have displayed a good safety profile in adults and more recently in infants. The immune responses to these molecules tend to be genogroup- and possibly genotype-specific and it is possible that several genotypes will be required in the vaccine formulation.

## 4. Future

### 4.1. NoV Vaccine Strategies: Coming of Age

Several population groups will eventually benefit from NoV vaccination—older individuals living in nursing homes where outbreaks of NoV gastroenteritis with severe consequences occur, including deaths; adult populations gathered in groups within relatively isolated areas; in strategic functions in which a NoV outbreak could produce a significant disruption (military personnel, peace corps, missions in isolated areas, such as space travel, high altitudes, mines, etc.). In a scenario of more widespread use, travelers may be able to consider a NoV vaccine if the vaccine demonstrated sufficient overall effectiveness in preventing traveler’s diarrhea.

Most importantly, NoV vaccines will be considered for use in children in order to further reduce diarrhea-associated hospitalizations and deaths. A highly effective NoV vaccine has the potential to reduce diarrhea-associated deaths by nearly 200,000 cases per year, with the greatest impact to be seen in the poorest regions of the world [24]. In these underprivileged areas, as well as in more privileged regions, diarrhea-associated hospitalizations could be reduced by nearly 3 million annually.

The implementation of pediatric vaccine programs will have several challenges given current vaccine developments. First, the fact that the most advanced vaccine candidates are to be administered intramuscularly complicates their implementation, as injections require a suitable infrastructure and human resources, that are more complicated and more expensive than those required by oral vaccines. Second, the willingness of parents to “jab” their infants to prevent a diarrheal pathogen in an already crowded schedule of injections, will also be a challenge. In the initial phases of implementation, it seems worthwhile to consider a NoV jab somewhere between 6 and 12 months of age, in order to not continue to crowd the 0–6 months schedule. The fact that NoV infections peak after 6 months of age could favor such an approach. Alternatively, and likely the optimal course of action, would be to include NoV in combination with another respiratory and/or enteric pathogen, for example Rotavirus. One common vaccine for two main diarrheal pathogens seems to be a reasonable avenue for the future. Data from Rotavirus suggest that both gut microbiota and host factors (e.g., secretor status) can influence the host’s response to a vaccine, and should be carefully assessed especially if a combined Rotavirus–NoV vaccination strategy is implemented.

### 4.2. Lessons from the “Coronavirus Pandemic”

The coronavirus pandemic has had important “indirect effects”, including an impressive reduction in respiratory and gastrointestinal infections throughout 2020 and at least the first half of 2021. In most countries with active respiratory virus surveillance, the “traditional” autumn–winter epidemic surge of respiratory syncytial virus (RSV), influenza, and other respiratory viruses was completely blunted [154]. For gastrointestinal viruses, epidemiological surveillance is in generally incomplete; nevertheless, a similar phenomenon was observed, particularly for NoV [155,156,157,158,159,160]. Several hypotheses can be generated to explain this significant decrease, including (i) school closures, leading to massive reductions in gatherings of children and children with adults; (ii) prolonged and intense quarantines; (iii) massive increases in hand hygiene procedures; (iv) global masking; (v) reduction in enteric pathogen testing capacity; and (vi) possible secondary effect of SARS-CoV2 infection (which can replicate on the gastrointestinal epithelium) on the predisposition to other enteric viruses, e.g., altering the gut microbiota composition or inducing an antiviral IFN response [157,160].

Thus, in what could be considered a natural global experiment, society has learned that mucosal pathogens, respiratory and enteric, causing significant disease every year, could eventually be controlled with important societal “restrictions”. Understandably, such restrictions have significant negative impacts, particularly school closures, that make them unfeasible in a non-pandemic situation. However, we can learn from them and partially apply measures that could aid in better control of these mucosal pathogens. Avoiding overcrowding in school classrooms, possibly by increasing and perfecting hybrid, presential/non-presential sessions, especially during harsh winter peaks in infections, could be a future consideration. Temporal, partial, or full school closures to control epidemic surges should also be on the list of possibilities. Hand and respiratory hygiene measures should remain and be enforced overtime, as there will be a natural tendency to relax these measures as the pandemic is forgotten. The regular use of face masks in reasonably eligible school aged children and adults will be a matter of discussion and debates in the near future. The potential positive effects of a reduction in pathogens spread through nasopharyngeal droplets will have to be weighted with the negative psychological effects of face covering, especially during the childhood years. Rigorous studies will be required to address these issues and provide evidence for reasonable future recommendations.

Resurgence of norovirus incidence after relaxation of non-pharmaceutical interventions related to SARS-CoV2 pandemic has been predicted [161]. Continued surveillance should be encouraged to allow adequate preparation for a potential increase in healthcare pressures beyond previous levels.

## Figures and Tables

**Table 1:** Selected recent publications (2018–2021) of the genotype distribution of human noroviruses.

Year Published	Years of Study	Location	NO. of Patients	Genogroups/Genotypes (%)	Comments	Reference
2021	2015–2020	Global	120,531	GII.4 (51)	Meta-analysis	[24]
				GII.3 (15)		
				GII.12 (5)		
2021	2010–2016	Brazil	251	GII.4[P31] (64)		[49]
				GII.17[P17] (6)		
				GII.1[P33] (6)		
2021	2017–2018	China	1500	GII.4 (44)	Outbreaks	[50]
				GII.17 (27)		
2020	2016–2017	Chile	174	GII.4 (35)	Surveillance Recombinants	[51]
				GII.6 (23)		
				GII.7 (12)		
2020	2015–2019	Indonesia	966	GII.[P31] (44)	HC	[34]
				GII.[P16] (37)		
2020	2009–2013	China	3134	GII.4 (50)	HP	[48]
				GII.17 (11)		
				GII.3 (8)		
2019	2014–2018	Brazil	61	GII.4 (19)	<5 years	[43]
				GII.6 (19)		
				GII.7 (19)		
2019	2016–2018	Qatar	177	GII.4 (62)	Children	[33]
				GII.2 (16)		
				GII.3 (14)		
2019	2014–2018	China	3422	GII.4 (72)	Outbreaks	[46]
				GII.3 (14)		
				GII.17 (8)		
2019	2010–2012	Bangladesh	819	GII.4 (33)	HC, <5 years	[52]
				GII.3 (13)		
				GII.6 (11)		
				GII.13 (11)		
2019	2012–2018	Japan	4588	GII.4 (22)	Surveillance	[53]
				GII.2 (15)		
				GII.17 (6)		
				GII.6 (4)		
2018	2015–2017	Thailand	1591	GII.4 (32)	Surveillance	[54]
				GII.17 (12)		
2018	2013–2015	Botswana	484	GII.4 (70)	HC, <5 years	[55]
				GII.2 (9)		
				GII.12 (9)		
				GI.9 (7)		
2018	2012–2013	Angola	343	GII.4 (20)	<5 years	[56]
				GII.6 (15)		
				GI.3 (12)		
				GII.10 (10)		

Abbreviations: HP—hospitalized patients; HC—hospitalized children.

**Table 2:** Vaccine candidates against Norovirus.

Vaccine Platform	Specific Antigens in the Vaccine	Expression System	Route and Schedule of Administration	Stage of Development	References
Virus-like particles	Bivalent GI.1 and GII.4	Baculovirus system	First delivered by intranasal route, and currently developed for intramuscular administration. Two doses separated by 30 days.	Clinical: Phase IIb completed; advancing to phase III trials in adults and children.	[134,135,136,137,138,139]
	Bivalent GI.1 and GII.4	Hansenula polymorpha	Intramuscular administration. Two to three doses separated by 28 days.	Clinical: Phase I completed; advancing to Phase II trials.	[139,140]
	Quadrivalent GI.1, GII.3, GII.4, GII.17	Pichia pastoris system	Intramuscular administration; doses under evaluation.	Clinical: Phase I/IIa ongoing	[139,141,142]
	Monovalent GII.4 VLPs	Plant expression system (tobacco, potato)	Oral and intranasal administration	Pre-clinical	[139,143,144]
	Quadrivalent GI.1, GI.3, GII.4, GII.12	Baculovirus expression system	Intramuscular administration.	Pre-clinical	[139]
	Trivalent Norovirus GI.3 and GII.4 and Rotavirus rVP6	Baculovirus expression system	Intramuscular administration.	Pre-clinical	[139,145,146]
	Bivalent Norovirus GII.4 and Enterovirus 71	Baculovirus expression system	Intraperitoneal administration.	Pre-clinical	[139,147]
P-particles	Monovalent GII.4	Baculovirus expression system	Intranasal administration.	Pre-clinical	[139,148]
	Monovalent GII.4 enhanced by adjuvant FlaB.	*E. coli* system	Intranasal and sublingual administration.	Pre-clinical	[139,149]
	Trivalent Norovirus, Hepatitis E and Astrovirus	*E. coli* system	Intranasal administration.	Pre-clinical	[139,150]
	Monovalent GII.2	Viral replicons in eukaryotic cell lines	Intranasal administration.	Pre-clinical	[139,151,152]
Adenovirus vector-based	Monovalent GI.1 or GII.4; or Bivalent GI.1 and GII.4 (co-expression with a double-stranded RNA adjuvant.	Human host cells	Oral administration; doses under evaluation.	Clinical: Phase I in adults completed; advancing to Phase II trials.	[139,153]

## References

[1] Yow M.D., Melnik J.L., Blsttner R.J., Stephenson W.B., Robinson N.M., Burkhardt M.A. (1970). The Association of Viruses and Bacteria with Infantile Diarrhea. Am. J. Epidemiol..

[2] Yalow R.S., Berson S.A. (1959). Assay of Plasma Insulin in Human Subjects by Immunological Methods. Nature.

[3] Dalrymple J.M., Vogel S.N., Teramoto A.Y., Russell P.K. (1973). Antigenic Components of Group A Arbovirus Virions. J. Virol..

[4] Ruska E. (1980). The Early Development of Electron Lenses and Electron Microscopy. Microsc. Acta. Suppl..

[5] Gordon I., Patterson P.R., Whitney E. (1956). Immunity in Volunteers Recovered from Non-Bacterial Gastroenteritis. J. Clin. Investig..

[6] Kojima S., Fukumi H., Kusama H., Yamamoto S., Suzuki S., Uchida T., Ishimaru T., Oka T., Kuretani K., Ohmura K. (1948). Studies on the Causative Agent of the Infectious Diarrhoea. Records of the Experiments on Human Volunteers. Jpn. Med. J..

[7] Kapikian A.Z., Wyatt R.G., Dolin R., Thornhill T.S., Kalica A.R., Chanock R.M. (1972). Visualization by Immune Electron Microscopy of a 27-Nm Particle Associated with Acute Infectious Nonbacterial Gastroenteritis. J. Virol..

[8] Kapikian A.Z. (2000). The Discovery of the 27-Nm Norwalk Virus: An Historic Perspective. J. Infect. Dis..

[9] Jung J., Grant T., Thomas D.R., Diehnelt C.W., Grigorieff N., Joshua-Tor L. (2019). High-Resolution Cryo-EM Structures of Outbreak Strain Human Norovirus Shells Reveal Size Variations. Proc. Natl. Acad. Sci. USA.

[10] Dolin R., Blacklow N.R., DuPont H., Buscho R.F., Wyatt R.G., Kasel J.A., Hornick R., Chanock R.M. (2016). Biological Properties of Norwalk Agent of Acute Infectious Nonbacterial Gastroenteritis. Exper. Biol. Med..

[11] Dolin R., Blacklow N.R., DuPont H., Formal S., Buscho R.F., Kasel J.A., Chames R.P., Hornick R., Chanock R.M. (1971). Transmission of Acute Infectious Nonbacterial Gastroenteritis to Volunteers by Oral Administration of Stool Filtrates. J. Infect. Dis..

[12] Greenberg H., Valdesuso J., Yolken R.H., Gangarosa E., Gary W., Wyatt R.G., Konno T., Suzuki H., Chanock R.M., Kapikian A.Z. (1979). Role of Norwalk Virus in Outbreaks of Nonbacterial Gastroenteritis. J. Infect. Dis..

[13] Greenberg H.B., Valdesuso J.R., Kalica A.R., Wyatt R.G., McAuliffe V.J., Kapikian A.Z., Chanock R.M. (1981). Proteins of Norwalk Virus. J. Virol..

[14] Jiang X., Wang M., Wang K., Estes M.K. (1993). Sequence and Genomic Organization of Norwalk Virus. Virology.

[15] Jiang X., Wang M., Graham D.Y., Estes M.K. (1992). Expression, Self-Assembly, and Antigenicity of the Norwalk Virus Capsid Protein. J. Virol..

[16] Hardy M.E., White L.J., Ball J.M., Estes M.K. (1995). Specific Proteolytic Cleavage of Recombinant Norwalk Virus Capsid Protein. J. Virol..

[17] Prasad B., Hardy M.E., Jiang X., Estes M.K. (1996). Structure of Norwalk Virus. Arch. Virol..

[18] Lambden P.R., Caul E.O., Ashley C.R., Clarke I.N. (1993). Sequence and genomic organization of a small round-structured (Norwalk-like) virus. Science.

[19] Carter M.J., Milton I.D., Meanger J., Bennett M., Gaskell R.M., Turner P.C. (1992). The complete nucleotide sequence of a feline calicivirus. Virology.

[20] Rinehart-Kim J., Zhong W.-M., Jiang X., Smith A.W., Matson D.O. (1999). Complete nucleotide sequence and genomic organization of a primate calicivirus, Pan-1. Arch. Virol..

[21] Zintz C., Bok K., Parada E., Barnes-Eley M., Berke T., Staat M.A., Azimi P., Jiang X., Matson D.O. (2005). Prevalence and Genetic Characterization of Caliciviruses among Children Hospitalized for Acute Gastroenteritis in the United States. Infect. Genet. Evol..

[22] Matson D.O. (2003). IV, 6. Calicivirus RNA Recombination. Perspect. Med. Virol..

[23] Jiang X., Espul C., Zhong W.M., Cuello H., Matson D.O. (1999). Characterization of a Novel Human Calicivirus That May Be a Naturally Occurring Recombinant. Arch. Virol..

[24] Farahmand M., Moghoofei M., Dorost A., Shoja Z., Ghorbani S., Kiani S.J., Khales P., Esteghamati A., Sayyahfar S., Jafarzadeh M. (2021). Global Prevalence and Genotype Distribution of Norovirus Infection in Children with Gastroenteritis: A Meta-Analysis on 6 Years of Research from 2015 to 2020. Rev. Med. Virol..

[25] Cannon J.L., Lopman B.A., Payne D.C., Vinjé J. (2019). Birth Cohort Studies Assessing Norovirus Infection and Immunity in Young Children: A Review. Clin. Infect. Dis..

[26] Lee H., Yoon Y. (2021). Etiological Agents Implicated in Foodborne Illness World. Food Sci. Anim. Resour..

[27] Queiros-Reis L., Lopes-João A., Mesquita J.R., Penha-Gonçalves C., Nascimento M.S.J. (2021). Norovirus Gastroenteritis Outbreaks in Military Units: A Systematic Review. BMJ Mil. Health.

[28] Troeger C., Forouzanfar M., Rao P.C., Khalil I., Brown A., Reiner R.C., Fullman N., Thompson R.L., Abajobir A., Ahmed M. (2017). Estimates of Global, Regional, and National Morbidity, Mortality, and Aetiologies of Diarrhoeal Diseases: A Systematic Analysis for the Global Burden of Disease Study 2015. Lancet Infect. Dis.

[29] Ahmed S.M., Hall A.J., Robinson A.E., Verhoef L., Premkumar P., Parashar U.D., Koopmans M., Lopman B.A. (2014). Global Prevalence of Norovirus in Cases of Gastroenteritis: A Systematic Review and Meta-Analysis. Lancet Infect. Dis..

[30] Burke R.M., Mattison C.P., Pindyck T., Dahl R.M., Rudd J., Bi D., Curns A.T., Parashar U., Hall A.J. (2021). Burden of Norovirus in the United States, as Estimated Based on Administrative Data: Updates for Medically Attended Illness and Mortality, 2001–2015. Clin. Infect. Dis..

[31] Nguyen G.T., Phan K., Teng I., Pu J., Watanabe T. (2017). A Systematic Review and Meta-Analysis of the Prevalence of Norovirus in Cases of Gastroenteritis in Developing Countries. Medicine.

[32] Mans J. (2019). Norovirus Infections and Disease in Lower-MiddleandLow-Income Countries, 1997–2018. Viruses.

[33] Mathew S., Alansari K., Smatti M.K., Zaraket H., Al Thani A.A., Yassine H.M. (2019). Epidemiological, Molecular, and Clinical Features of Norovirus Infections among Pediatric Patients in Qatar. Viruses.

[34] Wulandari P.S., Juniastuti, Wahyuni R.M., Amin M., Yamani L.N., Matondang M.Q.Y., Dinana Z., Soetjipto, Utsumi T., Shoji I. (2020). Predominance of Norovirus GI.4 from Children with Acute Gastroenteritis in Jambi, Indonesia, 2019. J. Med. Virol..

[35] Chen L., Xu D., Wu X., Liu G., Ji L. (2020). An Increasing Prevalence of Non-GII.4 Norovirus Genotypes in Acute Gastroenteritis Outbreaks in Huzhou, China, 2014–2018. Arch. Virol..

[36] Zhirakovskaia E.v., Tikunov A.Y., Bodnev S.A., Klemesheva V.v., Netesov S.v., Tikunova N.v. (2015). Molecular Epidemiology of Noroviruses Associated with Sporadic Gastroenteritis in Children in Novosibirsk, Russia, 2003–2012. J. Med. Virol..

[37] Kabue J.P., Meader E., Hunter P.R., Potgieter N. (2016). Human Norovirus Prevalence in Africa: A Review of Studies from 1990 to 2013. Trop. Med. Int. Health.

[38] Hardstaff J.L., Clough H.E., Lutje V., McIntyre K.M., Harris J.P., Garner P., O’Brien S.J. (2018). Foodborne and Food-Handler Norovirus Outbreaks: A Systematic Review. Foodborne Pathog. Dis..

[39] Mehlhorn H. (2015). WHO Estimates of the Global Burden of Foodborne Diseases: Foodborne Disease Burden Epidemiology Reference Group 2007–2015.

[40] Centers for Disease Control and Prevention (CDC) (2017). Surveillance for Foodborne Disease Outbreaks United States, 2017: Annual Report.

[41] Bert F., Scaioli G., Gualano M.R., Passi S., Specchia M.L., Cadeddu C., Viglianchino C., Siliquini R. (2014). Norovirus Outbreaks on Commercial Cruise Ships: A Systematic Review and New Targets for the Public Health Agenda. Food Environ. Virol..

[42] Chhabra P., De Graaf M., Parra G.I., Chan M.C.-W., Green K., Martella V., Wang Q., White P.A., Katayama K., Vennema H. (2019). Updated Classification of Norovirus Genogroups and Genotypes. J. Gen. Virol..

[43] Cantelli C.P., Da Silva M.F.M., Fumian T.M., Da Cunha D.C., Andrade J.D.S.R.D., Malta F.C., Junior S.D.S.E.M., Fialho A.M., De Moraes M.T.B., Brasil P. (2019). High Genetic Diversity of Noroviruses in Children from a Community-Based Study in Rio de Janeiro, Brazil, 2014-2018. Arch. Virol..

[44] Yi J., Wahl K., Sederdahl B.K., Jerris R.R., Kraft C.S., McCracken C., Gillespie S., Anderson E.J., Kirby A.E., Shane A.L. (2016). Molecular Epidemiology of Norovirus in Children and the Elderly in Atlanta, Georgia, United States. J. Med. Virol..

[45] Hoa Tran T.N., Trainor E., Nakagomi T., Cunliffe N.A., Nakagomi O. (2013). Molecular Epidemiology of Noroviruses Associated with Acute Sporadic Gastroenteritis in Children: Global Distribution of Genogroups, Genotypes and GII.4 Variants. J. Clin. Virol..

[46] Wang X., Wei Z., Guo J., Cai J., Chang H., Ge Y., Zeng M. (2019). Norovirus Activity and Genotypes in Sporadic Acute Diarrhea in Children in Shanghai During 2014–2018. Pediatric Infect. Dis. J..

[47] Lim K.L., Hewitt J., Sitabkhan A., Eden J.-S., Lun J., Levy A., Merif J., Smith D., Rawlinson W.D., White P.A. (2016). A Multi-Site Study of Norovirus Molecular Epidemiology in Australia and New Zealand, 2013–2014. PLoS ONE.

[48] Zhou H., Wang S., von Seidlein L., Wang X. (2019). The Epidemiology of Norovirus Gastroenteritis in China: Disease Burden and Distribution of Genotypes. Front. Med..

[49] Tinker R.J., da Costa A.C., Tahmasebi R., de Pádua Milagres F.A., dos Santos Morais V., Pandey R.P., José-Abrego A., Brustulin R., Rodrigues Teles M.d.A., Cunha M.S. (2021). Norovirus Strains in Patients with Acute Gastroenteritis in Rural and Low-Income Urban Areas in Northern Brazil. Arch. Virol..

[50] Sun C., Zhao Y., Wang G., Huang D., He H., Sai L. (2020). Molecular Epidemiology of GII Noroviruses in Outpatients with Acute Gastroenteritis in Shandong Province, China. Arch. Virol..

[51] Lucero Y., Lagomarcino A.J., Espinoza M., Kawakami N., Mamani N., Huerta N., del Canto F., Farfán M., Sawaguchi Y., George S. (2020). Norovirus Compared to Other Relevant Etiologies of Acute Gastroenteritis among Families from a Semirural County in Chile. Int. J. Infect. Dis..

[52] Hossain M.E., Rahman R., Ali S.I., Islam M.M., Rahman M.Z., Ahmed S., Faruque A.S.G., Barclay L., Vinjé J., Rahman M. (2019). Epidemiologic and Genotypic Distribution of Noroviruses Among Children with Acute Diarrhea and Healthy Controls in a Low-Income Rural Setting. Clin. Infect. Dis..

[53] Motoya T., Umezawa M., Saito A., Goto K., Doi I., Fukaya S., Nagata N., Ikeda Y., Okayama K., Aso J. (2019). Variation of Human Norovirus GII Genotypes Detected in Ibaraki, Japan, during 2012–2018. Gut Pathog..

[54] Thanusuwannasak T., Puenpa J., Chuchaona W., Vongpunsawad S., Poovorawan Y. (2018). Emergence of Multiple Norovirus Strains in Thailand, 2015–2017. Infect. Genet. Evol. J. Mol. Epidemiol. Evol. Genet. Infect. Dis..

[55] Makhaola K., Moyo S., Lechiile K., Goldfarb D.M., Kebaabetswe L.P. (2018). Genetic and Epidemiological Analysis of Norovirus from Children with Gastroenteritis in Botswana, 2013–2015. BMC Infect. Dis..

[56] Esteves A., Nordgren J., Tavares C., Fortes F., Dimbu R., Saraiva N., Istrate C. (2018). Genetic Diversity of Norovirus in Children under 5 Years of Age with Acute Gastroenteritis from Angola. Epidemiol. Infect..

[57] Ettayebi K., Crawford S.E., Murakami K., Broughman J.R., Karandikar U., Tenge V., Neill F.H., Blutt S.E., Zeng X.-L., Qu L. (2016). Replication of Human Noroviruses in Stem Cell-Derived Human Enteroids. Science.

[58] Murakami K., Tenge V.R., Karandikar U.C., Lin S.-C., Ramani S., Ettayebi K., Crawford S.E., Zeng X.-L., Neill F.H., Ayyar B.V. (2020). Bile Acids and Ceramide Overcome the Entry Restriction for GII.3 Human Norovirus Replication in Human Intestinal Enteroids. Proc. Natl. Acad. Sci. USA.

[59] De Graaf M., van Beek J., Koopmans M.P.G. (2016). Human Norovirus Transmission and Evolution in a Changing World. Nat. Rev. Microbiol..

[60] Keswick B.H., Satterwhite T.K., Johnson P.C., DuPont H.L., Secor S.L., Bitsura J.A., Gary G.W., Hoff J.C. (1985). Inactivation of Norwalk Virus in Drinking Water by Chlorine. Appl. Environ. Microbiol..

[61] Ahmed K., Dony J.J.F., Mori D., Haw L.Y., Giloi N., Jeffree M.S., Iha H. (2020). An Outbreak of Gastroenteritis by Emerging Norovirus GII.2[P16] in a Kindergarten in Kota Kinabalu, Malaysian Borneo. Sci. Rep..

[62] Matsuyama R., Miura F., Nishiura H. (2017). The Transmissibility of Noroviruses: Statistical Modeling of Outbreak Events with Known Route of Transmission in Japan. PLoS ONE.

[63] Gaythorpe K.A.M., Trotter C.L., Lopman B., Steele M., Conlan A.J.K. (2018). Norovirus Transmission Dynamics: A Modelling Review. Epidemiol. Infect..

[64] Rockx B., de Wit M., Vennema H., Vinjé J., de Bruin E., van Duynhoven Y., Koopmans M. (2002). Natural History of Human Calicivirus Infection: A Prospective Cohort Study. Clin. Infect. Dis..

[65] Siebenga J.J., Beersma M.F.C., Vennema H., van Biezen P., Hartwig N.J., Koopmans M. (2008). High Prevalence of Prolonged Norovirus Shedding and Illness among Hospitalized Patients: A Model for In Vivo Molecular Evolution. J. Infect. Dis..

[66] Chan M.C.W., Sung J.J.Y., Lam R.K.Y., Chan P.K.S., Lee N.L.S., Lai R.W.M., Leung W.K. (2006). Fecal Viral Load and Norovirus-Associated Gastroenteritis. Emerg. Infect. Dis..

[67] Koo H.L., Dupont H.L. (2009). Noroviruses as a Potential Cause of Protracted and Lethal Disease in Immunocompromised Patients. Clin. Infect. Dis..

[68] Miura F., Matsuyama R., Nishiura H. (2018). Estimating the Asymptomatic Ratio of Norovirus Infection During Foodborne Outbreaks with Laboratory Testing in Japan. J. Epidemiol..

[69] Riera-Montes M., O’Ryan M., Verstraeten T. (2018). Norovirus and Rotavirus Disease Severity in Children: Systematic Review and Meta-Analysis. Pediatric Infect. Dis. J..

[70] Rahman M., Rahman R., Nahar S., Hossain S., Ahmed S., Faruque A.S.G., Azim T. (2016). Norovirus Diarrhea in Bangladesh, 2010-2014: Prevalence, Clinical Features, and Genotypes. J. Med. Virol..

[71] Lindsay L., Wolter J., De Coster I., Van Damme P., Verstraeten T. (2015). A Decade of Norovirus Disease Risk among Older Adults in Upper-Middle and High Income Countries: A Systematic Review. BMC Infect. Dis..

[72] Brown L.-A.K., Ruis C., Clark I., Roy S., Brown J.R., Albuquerque A.S., Patel S.Y., Miller J., Karim M.Y., Dervisevic S. (2019). A Comprehensive Characterization of Chronic Norovirus Infection in Immunodeficient Hosts. J. Allergy Clin. Immunol..

[73] Woodward J., GkraniaKlotsas E. (2017). Chronic Norovirus Infection and Common Variable Immunodeficiency. Clin. Exp. Immunol..

[74] Brown J.R., Roy S., Tutill H., Williams R., Breuer J. (2017). Super-Infections and Relapses Occur in Chronic Norovirus Infections. J. Clin. Virol..

[75] Stuart R.L., Tan K., Mahar J., Kirkwood C.D., Ramsden C.A., Andrianopoulos N., Jolley D., Bawden K., Doherty R., Kotsanas D. (2010). An Outbreak of Necrotizing Enterocolitis Associated with Norovirus Genotype GII.3. Pediatric Infect. Dis. J..

[76] Turcios-Ruiz R.M., Axelrod P., John K.S., Bullitt E., Donahue J., Robinson N., Friss H.E. (2008). Outbreak of Necrotizing Enterocolitis Caused by Norovirus in a Neonatal Intensive Care Unit. J. Pediatrics.

[77] Khan R.R., Lawson A.D., Minnich L.L., Martin K., Nasir A., Emmett M.K., Welch C.A., Udall J.N. (2009). Gastrointestinal Norovirus Infection Associated with Exacerbation of Inflammatory Bowel Disease. J. Pediatric Gastroenterol. Nutr..

[78] Tarris G., De Rougemont A., Charkaoui M., Michiels C., Martin L., Belliot G. (2021). Enteric Viruses and Inflammatory Bowel Disease. Viruses.

[79] Porter C.K., Faix D.J., Shiau D., Espiritu J., Espinosa B.J., Riddle M.S. (2012). Postinfectious Gastrointestinal Disorders Following Norovirus Outbreaks. Clin. Infect. Dis..

[80] Zanini B., Ricci C., Bandera F., Caselani F., Magni A., Laronga A.M., Lanzini A. (2012). Incidence of Post-Infectious Irritable Bowel Syndrome and Functional Intestinal Disorders Following a Water-Borne Viral Gastroenteritis Outbreak. Am. J. Gastroenterol..

[81] Fujita Y., Kohira R., Fuchigami T., Mugishima H. (2009). Detection of Rotavirus RNA and Antigens in Serum and Cerebrospinal Fluid Samples from Diarrheic Children with Seizures. Jpn. J. Infect. Dis..

[82] Reymão T.K.A., Fumian T.M., Justino M.C.A., Hernandez J.M., Bandeira R.S., Lucena M.S.S., Teixeira D.M., Farias F.P., Silva L.D., Linhares A.C. (2018). Norovirus RNA in Serum Associated with Increased Fecal Viral Load in Children: Detection, Quantification and Molecular Analysis. PLoS ONE.

[83] Ho C.L.T., Oligbu O., Asaid F., Oligbu G. (2020). Does Norovirus Induce Acute Hepatitis?. AIMS Public Health.

[84] Shima T., Okumura A., Kurahashi H., Numoto S., Abe S., Ikeno M., Shimizu T. (2019). A Nationwide Survey of Norovirus-Associated Encephalitis/Encephalopathy in Japan. Brain Dev..

[85] Ueda H., Tajiri H., Kimura S., Etani Y., Hosoi G., Maruyama T., Noma H., Kusumoto Y., Takano T., Baba Y. (2015). Clinical Characteristics of Seizures Associated with Viral Gastroenteritis in Children. Epilepsy Res..

[86] Hu M.-H., Lin K.-L., Wu C.-T., Chen S.-Y., Huang G.-S. (2017). Clinical Characteristics and Risk Factors for Seizures Associated With Norovirus Gastroenteritis in Childhood. J. Child Neurol..

[87] van Dycke J., Ny A., Conceição-Neto N., Maes J., Hosmillo M., Cuvry A., Goodfellow I., Nogueira T.C., Verbeken E., Matthijnssens J. (2019). A Robust Human Norovirus Replication Model in Zebrafish Larvae. PLoS Pathog..

[88] Wobus C.E., Thackray L.B., Virgin H.W. (2006). Murine Norovirus: A Model System to Study Norovirus Biology and Pathogenesis. J. Virol..

[89] Lee S., Wilen C.B., Orvedahl A., McCune B.T., Kim K.-W., Orchard R.C., Peterson S.T., Nice T., Baldridge M.T., Virgin H.W. (2017). Norovirus Cell Tropism Is Determined by Combinatorial Action of a Viral Non-Structural Protein and Host Cytokine. Cell Host Microbe.

[90] Orchard R.C., Wilen C.B., Doench J.G., Baldridge M.T., McCune B.T., Lee Y.-C.J., Lee S., Pruett-Miller S.M., Nelson C.A., Fremont D.H. (2016). Discovery of a Proteinaceous Cellular Receptor for a Norovirus. Science.

[91] Snowden J.S., Hurdiss D.L., Adeyemi O.O., Ranson N.A., Herod M.R., Stonehouse N.J. (2020). Dynamics in the Murine Norovirus Capsid Revealed by High-Resolution Cryo-EM. PLoS Biol..

[92] Smith H.Q., Smith T.J. (2019). The Dynamic Capsid Structures of the Noroviruses. Viruses.

[93] Sherman M.B., Williams A.N., Smith H.Q., Nelson C., Wilen C.B., Fremont D.H., Virgin H.W., Smith T.J. (2019). Bile Salts Alter the Mouse Norovirus Capsid Conformation: Possible Implications for Cell Attachment and Immune Evasion. J. Virol..

[94] Nelson C.A., Wilen C.B., Dai Y.N., Orchard R.C., Kim A.S., Stegeman R.A., Hsieh L.L., Smith T.J., Virgin H.W., Fremont D.H. (2018). Structural Basis for Murine Norovirus Engagement of Bile Acids and the CD300lf Receptor. Proc. Natl. Acad. Sci. USA.

[95] Taube S., Perry J.W., McGreevy E., Yetming K., Perkins C., Henderson K., Wobus C.E. (2012). Murine Noroviruses Bind Glycolipid and Glycoprotein Attachment Receptors in a Strain-Dependent Manner. J. Virol..

[96] Creutznacher R., Maass T., Ogrissek P., Wallmann G., Feldmann C., Peters H., Lingemann M., Taube S., Peters T., Mallagaray A. (2021). NMR Experiments Shed New Light on Glycan Recognition by Human and Murine Norovirus Capsid Proteins. Viruses.

[97] Karandikar U.C., Crawford S.E., Ajami N.J., Murakami K., Kou B., Ettayebi K., Papanicolaou G.A., Jongwutiwes U., Perales M.A., Shia J. (2016). Detection of Human Norovirus in Intestinal Biopsies from Immunocompromised Transplant Patients. J. Gen. Virol..

[98] Green K.Y., Kaufman S.S., Nagata B.M., Chaimongkol N., Kim D.Y., Levenson E.A., Tin C.M., Yardley A.B., Johnson J.A., Barletta A.B.F. (2020). Human Norovirus Targets Enteroendocrine Epithelial Cells in the Small Intestine. Nat. Commun..

[99] Chan J.C.M., Mohammad K.N., Zhang L.Y., Wong S.H., Chan M.C.W. (2021). Targeted Profiling of Immunological Genes during Norovirus Replication in Human Intestinal Enteroids. Viruses.

[100] Green K.Y. (2016). Editorial Commentary: Noroviruses and B Cells. Clin. Infect. Dis..

[101] Jones M.K., Watanabe M., Zhu S., Graves C.L., Keyes L.R., Grau K.R., Gonzalez-Hernandez M.B., Iovine N.M., Wobus C.E., Vinjé J. (2014). Enteric Bacteria Promote Human and Mouse Norovirus Infection of B Cells. Science.

[102] Nordgren J., Svensson L. (2019). Genetic Susceptibility to Human Norovirus Infection: An Update. Viruses.

[103] Yamakawa N., Vanbeselaere J., Chang L.Y., Yu S.Y., Ducrocq L., Harduin-Lepers A., Kurata J., Aoki-Kinoshita K.F., Sato C., Khoo K.H. (2018). Systems Glycomics of Adult Zebrafish Identifies Organ-Specific Sialylation and Glycosylation Patterns. Nat. Commun..

[104] Kilic T., Koromyslova A., Hansman G.S. (2019). Structural Basis for Human Norovirus Capsid Binding to Bile Acids. J. Virol..

[105] Hassan E., Baldridge M.T. (2019). Norovirus Encounters in the Gut: Multifaceted Interactions and Disease Outcomes. Mucosal Immunol..

[106] Newman K.L., Moe C.L., Kirby A.E., Flanders W.D., Parkos C.A., Leon J.S. (2015). Human Norovirus Infection and the Acute Serum Cytokine Response. Clin. Exp. Immunol..

[107] Newman K.L., Moe C.L., Kirby A.E., Flanders W.D., Parkos C.A., Leon J.S. (2016). Norovirus in Symptomatic and Asymptomatic Individuals: Cytokines and Viral Shedding. Clin. Exp. Immunol..

[108] Lin S.C., Qu L., Ettayebi K., Crawford S.E., Blutt S.E., Robertson M.J., Zeng X.L., Tenge V.R., Ayyar B.V., Karandikar U.C. (2020). Human Norovirus Exhibits Strain-Specific Sensitivity to Host Interferon Pathways in Human Intestinal Enteroids. Proc. Natl. Acad. Sci. USA.

[109] Newman K.L., Leon J.S. (2015). Norovirus Immunology: Of Mice and Mechanisms. Eur. J. Immunol..

[110] Chachu K.A., LoBue A.D., Strong D.W., Baric R.S., Virgin H.W. (2008). Immune Mechanisms Responsible for Vaccination against and Clearance of Mucosal and Lymphatic Norovirus Infection. PLoS Pathog..

[111] Nice T.J., Baldridge M.T., McCune B.T., Norman J.M., Lazear H.M., Artyomov M., Diamond M.S., Virgin H.W. (2015). Interferon-λ Cures Persistent Murine Norovirus Infection in the Absence of Adaptive Immunity. Science.

[112] Alvarado G., Salmen W., Ettayebi K., Hu L., Sankaran B., Estes M.K., Venkataram Prasad B.v., Crowe J.E. (2021). Broadly Cross-Reactive Human Antibodies That Inhibit Genogroup I and II Noroviruses. Nat. Commun..

[113] Pattekar A., Mayer L.S., Lau C.W., Liu C., Palko O., Bewtra M., Consortium H.P.A.P., Lindesmith L.C., Brewer-Jensen P.D., Baric R.S. (2021). Norovirus-Specific CD8+ T Cell Responses in Human Blood and Tissues. Cell. Mol. Gastroenterol. Hepatol..

[114] Saif M.A., Bonney D.K., Bigger B., Forsythe L., Williams N., Page J., Babiker Z.O., Guiver M., Turner A.J., Hughes S. (2011). Chronic Norovirus Infection in Pediatric Hematopoietic Stem Cell Transplant Recipients: A Cause of Prolonged Intestinal Failure Requiring Intensive Nutritional Support. Pediatric Transplant..

[115] Axelrad J.E., Cadwell K.H., Colombel J.-F., Shah S.C. (2020). Systematic Review: Gastrointestinal Infection and Incident Inflammatory Bowel Disease. Aliment. Pharmacol. Ther..

[116] Cadwell K., Patel K.K., Maloney N.S., Liu T.-C., Ng A.C., Storer C., Head R.D., Xavier R., Stappenbeck T.S., Virgin H.W. (2010). Virus-plus-Susceptibility Gene Interaction Determines Crohn’s Disease Gene Atg16L1 Phenotypes in Intestine. Cell.

[117] Baldridge M.T., Nice T.J., McCune B.T., Yokoyama C.C., Kambal A., Wheadon M., Diamond M.S., Ivanova Y., Artyomov M., Virgin H.W. (2015). Commensal Microbes and Interferon-λ Determine Persistence of Enteric Murine Norovirus Infection. Science.

[118] Turula H., Bragazzi Cunha J., Mainou B.A., Ramakrishnan S.K., Wilke C.A., Gonzalez-Hernandez M.B., Pry A., Fava J., Bassis C.M., Edelman J. (2018). Natural Secretory Immunoglobulins Promote Enteric Viral Infections. J. Virol..

[119] Wilen C.B., Lee S., Hsieh L.L., Orchard R.C., Desai C., Hykes B.L., McAllaster M.R., Balce D.R., Feehley T., Brestoff J.R. (2018). Tropism for Tuft Cells Determines Immune Promotion of Norovirus Pathogenesis. Science.

[120] Li D., Breiman A., Le Pendu J., Uyttendaele M. (2015). Binding to Histo-Blood Group Antigen-Expressing Bacteria Protects Human Norovirus from Acute Heat Stress. Front. Microbiol..

[121] Lee H., Ko G. (2016). Antiviral Effect of Vitamin A on Norovirus Infection via Modulation of the Gut Microbiome. Sci. Rep..

[122] Lee W., Kim M., Lee S.-H., Jung H.-G., Oh J.-W. (2018). Prophylactic Efficacy of Orally Administered Bacillus Poly-γ-Glutamic Acid, a Non-LPS TLR4 Ligand, against Norovirus Infection in Mice. Sci. Rep..

[123] Rodríguez-Díaz J., García-Mantrana I., Vila-Vicent S., Gozalbo-Rovira R., Buesa J., Monedero V., Collado M.C. (2017). Relevance of Secretor Status Genotype and Microbiota Composition in Susceptibility to Rotavirus and Norovirus Infections in Humans. Sci. Rep..

[124] Patin N.V., Peña-Gonzalez A., Hatt J.K., Moe C., Kirby A., Konstantinidis K.T. (2020). The Role of the Gut Microbiome in Resisting Norovirus Infection as Revealed by a Human Challenge Study. mBio.

[125] Hickman D., Jones M.K., Zhu S., Kirkpatrick E., Ostrov D.A., Wang X., Ukhanova M., Sun Y., Mai V., Salemi M. (2014). The Effect of Malnutrition on Norovirus Infection. mBio.

[126] Nelson A.M., Elftman M.D., Pinto A.K., Baldridge M., Hooper P., Kuczynski J., Petrosino J.F., Young V.B., Wobus E.C. (2013). Murine Norovirus Infection Does Not Cause Major Disruptions in the Murine Intestinal Microbiota. Microbiome.

[127] Nelson A.M., Walk S.T., Taube S., Taniuchi M., Houpt E.R., Wobus C., Young V.B. (2012). Disruption of the Human Gut Microbiota Following Norovirus Infection. PLoS ONE.

[128] Chen S.-Y., Tsai C.-N., Lee Y.-S., Lin C.-Y., Huang K.-Y., Chao H.-C., Lai M.-W., Chiu C.-H. (2017). Intestinal Microbiome in Children with Severe and Complicated Acute Viral Gastroenteritis. Sci. Rep..

[129] Mathew S., Smatti M.K., al Ansari K., Nasrallah G.K., al Thani A.A., Yassine H.M. (2019). Mixed Viral-Bacterial Infections and Their Effects on Gut Microbiota and Clinical Illnesses in Children. Sci. Rep..

[130] Xiong L., Li Y., Li J., Yang J., Shang L., He X., Liu L., Luo Y., Xie X. (2021). Intestinal Microbiota Profiles in Infants with Acute Gastroenteritis Caused by Rotavirus and Norovirus Infection: A Prospective Cohort Study. Int. J. Infect. Dis..

[131] Santos-Ferreira N., van Dycke J., Neyts J., Rocha-Pereira J. (2021). Current and Future Antiviral Strategies to Tackle Gastrointestinal Viral Infections. Microorganisms.

[132] Hallowell B.D., Parashar U.D., Hall A.J. (2018). Epidemiologic Challenges in Norovirus Vaccine Development. Hum. Vaccines Immunother..

[133] Lee B.E., Pang X.-L. (2014). One More Step toward Understanding the Immune Response to Norovirus. J. Infect. Dis..

[134] Atmar R.L., Baehner F., Cramer J.P., Song E., Borkowski A., Mendelman P.M., Al-Ibrahim M.S., Bernstein D.L., Brandon D.M., Chu L. (2016). Rapid Responses to 2 Virus-Like Particle Norovirus Vaccine Candidate Formulations in Healthy Adults: A Randomized Controlled Trial. J. Infect. Dis..

[135] Sundararajan A., Sangster M.Y., Frey S., Atmar R.L., Chen W.H., Ferreira J., Bargatze R., Mendelman P.M., Treanor J.J., Topham D.J. (2015). Robust Mucosal-Homing Antibody-Secreting B Cell Responses Induced by Intramuscular Administration of Adjuvanted Bivalent Human Norovirus-like Particle Vaccine. Vaccine.

[136] Treanor J.J., Atmar R.L., Frey S.E., Gormley R., Chen W.H., Ferreira J., Goodwin R., Borkowski A., Clemens R., Mendelman P.M. (2014). A Novel Intramuscular Bivalent Norovirus Virus-like Particle Vaccine Candidate--Reactogenicity, Safety, and Immunogenicity in a Phase 1 Trial in Healthy Adults. J. Infect. Dis..

[137] Masuda T., Lefevre I., Mendelman P., Sherwood J., Bizjajeva S., Borkowski A. (2018). 2276. Immunogenicity of Takeda’s Bivalent Virus-Like Particle (VLP) Norovirus Vaccine (NoV) Candidate in Children From 6 Months up to 4 Years of Age. Open Forum Infect. Dis..

[138] Leroux-Roels G., Cramer J.P., Mendelman P.M., Sherwood J., Clemens R., Aerssens A., De Coster I., Borkowski A., Baehner F., Van Damme P. (2018). Safety and Immunogenicity of Different Formulations of Norovirus Vaccine Candidate in Healthy Adults: A Randomized, Controlled, Double-Blind Clinical Trial. J. Infect. Dis..

[139] Home—ClinicalTrials.Gov. https://clinicaltrials.gov/.

[140] Zhang J., Tang F., Zhang X., Ma Z., Liu Z., Liang Y., Li Q. (2017). Physicochemical and Immunological Characterization of Two Forms of Recombinant Norovirus GⅡ.4 Virus-like Particles Assembled in Hansenula Polymorpha. Chin. J. Microbiol. Immunol..

[141] Tomé-Amat J., Fleischer L., Parker S.A., Bardliving C.L., Batt C.A. (2014). Secreted Production of Assembled Norovirus Virus-like Particles from Pichia Pastoris. Microb. Cell Factories.

[142] Parker S.A., Maloy M.H., Tome-Amat J., Bardliving C.L., Batt C.A., Lanz K.J., Olesberg J.T., Arnold M.A. (2016). Optimization of Norovirus Virus-like Particle Production in Pichia Pastoris Using a Real-Time near-Infrared Bioprocess Monitor. Biotechnol. Prog..

[143] Mathew L.G., Herbst-Kralovetz M., Mason H.S. (2014). Norovirus Narita 104 Virus-like Particles Expressed in Nicotiana Benthamiana Induce Serum and Mucosal Immune Responses. BioMed Res. Int..

[144] Santi L., Batchelor L., Huang Z., Hjelm B., Kilbourne J., Arntzen C.J., Chen Q., Mason H.S. (2008). An Efficient Plant Viral Expression System Generating Orally Immunogenic Norwalk Virus-like Particles. Vaccine.

[145] Malm M., Tamminen K., Lappalainen S., Vesikari T., Blazevic V. (2016). Rotavirus Recombinant VP6 Nanotubes Act as an Immunomodulator and Delivery Vehicle for Norovirus Virus-Like Particles. J. Immunol. Res..

[146] Tamminen K., Lappalainen S., Huhti L., Vesikari T., Blazevic V. (2013). Trivalent Combination Vaccine Induces Broad Heterologous Immune Responses to Norovirus and Rotavirus in Mice. PLoS ONE.

[147] Wang X., Ku Z., Dai W., Chen T., Ye X., Zhang C., Zhang Y., Liu Q., Jin X., Huang Z. (2015). A Bivalent Virus-like Particle Based Vaccine Induces a Balanced Antibody Response against Both Enterovirus 71 and Norovirus in Mice. Vaccine.

[148] Kocher J., Bui T., Giri-Rachman E., Wen K., Li G., Yang X., Liu F., Tan M., Xia M., Zhong W. (2014). Intranasal P Particle Vaccine Provided Partial Cross-Variant Protection against Human GII.4 Norovirus Diarrhea in Gnotobiotic Pigs. J. Virol..

[149] Verma V., Tan W., Puth S., Cho K.-O., Lee S.E., Rhee J.H. (2016). Norovirus (NoV) Specific Protective Immune Responses Induced by Recombinant P Dimer Vaccine Are Enhanced by the Mucosal Adjuvant FlaB. J. Transl. Med..

[150] Xia M., Wei C., Wang L., Cao D., Meng X.-J., Jiang X., Tan M. (2016). A Trivalent Vaccine Candidate against Hepatitis E Virus, Norovirus, and Astrovirus. Vaccine.

[151] Ma Y., Li J. (2011). Vesicular Stomatitis Virus as a Vector to Deliver Virus-like Particles of Human Norovirus: A New Vaccine Candidate against an Important Noncultivable Virus. J. Virol..

[152] Kim S.-H., Chen S., Jiang X., Green K.Y., Samal S.K. (2014). Newcastle Disease Virus Vector Producing Human Norovirus-like Particles Induces Serum, Cellular, and Mucosal Immune Responses in Mice. J. Virol..

[153] Kim L., Liebowitz D., Lin K., Kasparek K., Pasetti M.F., Garg S.J., Gottlieb K., Trager G., Tucker S.N. (2018). Safety and Immunogenicity of an Oral Tablet Norovirus Vaccine, a Phase I Randomized, Placebo-Controlled Trial. JCI Insight.

[154] Groves H.E., Piché-Renaud P.-P., Peci A., Farrar D.S., Buckrell S., Bancej C., Sevenhuysen C., Campigotto A., Gubbay J.B., Morris S.K. (2021). The Impact of the COVID-19 Pandemic on Influenza, Respiratory Syncytial Virus, and Other Seasonal Respiratory Virus Circulation in Canada: A Population-Based Study. Lancet Reg. Health. Am..

[155] Ondrikova N., Clough H.E., Douglas A., Iturriza-Gomara M., Larkin L., Vivancos R., Harris J.P., Cunliffe N.A. (2021). Differential Impact of the COVID-19 Pandemic on Laboratory Reporting of Norovirus and Campylobacter in England: A Modelling Approach. PLoS ONE.

[156] Fukuda Y., Tsugawa T., Nagaoka Y., Ishii A., Nawa T., Togashi A., Kunizaki J., Hirakawa S., Iida J., Tanaka T. (2021). Surveillance in Hospitalized Children with Infectious Diseases in Japan: Pre- and Post-Coronavirus Disease 2019. J. Infect. Chemother..

[157] Eigner U., Verstraeten T., Weil J. (2021). Decrease in Norovirus Infections in Germany Following COVID-19 Containment Measures. J. Infect..

[158] Lennon R.P., Griffin C., Miller E.L., Dong H., Rabago D., Zgierska A.E. (2020). Norovirus Infections Drop 49% in the United States with Strict COVID-19 Public Health Interventions. Acta Med. Acad..

[159] Bruggink L.D., Garcia-Clapes A., Tran T., Druce J.D., Thorley B.R. (2021). Decreased Incidence of Enterovirus and Norovirus Infections during the COVID-19 Pandemic, Victoria, Australia, 2020. Commun. Dis. Intell..

[160] Kraay A.N.M., Han P., Kambhampati A.K., Wikswo M.E., Mirza S.A., Lopman B.A. (2021). Impact of Nonpharmaceutical Interventions for Severe Acute Respiratory Syndrome Coronavirus 2 on Norovirus Outbreaks: An Analysis of Outbreaks Reported By 9 US States. J. Infect. Dis..

[161] O’Reilly K.M., Sandman F., Allen D., Jarvis C.I., Gimma A., Douglas A., Larkin L., Wong K.L.M., Baguelin M., Baric R.S. (2021). Predicted Norovirus Resurgence in 2021–2022 Due to the Relaxation of Nonpharmaceutical Interventions Associated with COVID-19 Restrictions in England: A Mathematical Modeling Study. BMC Med..

